# An empirical investigation of the impact of smoking on body weight using an endogenous treatment effects model approach: the role of food consumption patterns

**DOI:** 10.1186/s12937-018-0408-0

**Published:** 2018-11-05

**Authors:** Elena Raptou, Georgios Papastefanou

**Affiliations:** 1 Department of Agricultural Development, Laboratory of Management and Marketing, Democritus University of Thrace, Orestiada, Greece; 2 GESIS-Leibniz Institute for the Social Sciences, Mannheim, Germany

**Keywords:** BMI, Smoking behaviour, Food consumption patterns, Obesity, Endogeneity bias, Endogenous treatment effects model, Ordered outcome

## Abstract

**Background:**

This study explored the influence of cigarette smoking and food consumption patterns on BMI after adjusting for various socio-demographic characteristics. Since weight-based stereotypes may have an impact on smoking behaviour and both obesity and smoking have been associated with detrimental health effects, an interdependency between them is quite possible.

**Methods:**

Cross-sectional data were collected via a formal standardized questionnaire administered in personal interviews and two additional self-completion questionnaires from a random sample of 3471 German adults. The empirical framework considered potential endogeneity between smoking and body weight by employing an endogenous treatment effects model with an ordered outcome. The estimations derived from the endogenous treatment effects approach were also compared to the univariate ordered probit model results.

**Results:**

Our findings showed that ignoring potential endogeneity may affect both the statistical significance of the smoking estimate and the direction of the influence of smoking on BMI. Smoking was positively associated with BMI in both male (*β* = 1.236, *p* < 0.01) and female (*β* = 0.634, *p* < 0.10) participants. Smokers presented a 23.1% higher risk of obesity and a 24.3% lower likelihood of being within a healthy weight range. Male smokers also appeared to have a considerably augmented probability of being obese compared to their female counterparts (23.6% vs 15.1%). The relationship between smoking and BMI may be attributed to dietary practices, since smoking was correlated with poor dietary habits characterized by the frequent intake of more energy-dense foods (meat products and white-toasted bread) and less frequent consumption of healthy food items, such as whole-grain bread, vegetables and fruits. Concerning the impact of eating habits on body weight, frequent consumption of meat products and confectionery was found to have a direct association with BMI in both genders. Furthermore, white-toasted bread consumption was negatively linked with body weight in males (β = − 0.337, *p* < 0.01).

**Conclusions:**

Our findings raised questions about the general belief that smoking is an effective weight control tool. Health interventions should be oriented toward a simultaneous decrease in smoking and obesity, since both behaviours seem to be interdependent. Nutrition programmes should also be designed according to the characteristics of different target groups in order to promote a healthy lifestyle.

## Introduction

Obesity has been correlated with a host of adverse health effects, such as cardiovascular disease, type-2 diabetes, obesity-related cancers and osteoarthritis [1, 2]. There is also a wide range of psychological burdens concomitant with obesity. Obese people are highly stigmatized and appear to be more vulnerable to depression, body dissatisfaction, low self-esteem, and eating and psychiatric disorders [3–5].

Obesity stereotypes and stigma may have an impact on smoking behaviour, and there has been a growing interest in understanding the relationship between smoking and body weight [6]. Given the well-documented association between smoking cessation and weight gain [7, 8], susceptibility to post-cessation weight gain has been reported as a potential barrier to quitting smoking, especially among women [9]. There is a substantial body of evidence showing that smokers may use smoking to control their appetite and weight and that smoking rates and the frequency of cigarette consumption may be associated with subjective beliefs and exaggerated expectations about the efficacy of smoking as a weight-control strategy [10, 11]. In addition, individuals may consciously decide to smoke in order to counteract the effects of overeating. This appears to be more prominent in female smokers, by whom smoking is widely used to restrict food intake, and there is a widespread belief that smoking will help compensate for the effects of overindulging in food cravings [11].

It is widely perceived that smoking can decrease body weight by reducing appetite and caloric intake, improving metabolism, and leading to lower fat accumulation due to the impact of nicotine on the brain’s regulation of appetite and energy expenditure [12, 13]. On the other hand, cigarette consumption can reduce physical activity by restricting respiratory function, and hence lead to an increase in body weight [13, 14]. Therefore, biological pathways suggest that there is no consensus on the interrelationship between smoking and body weight.

A growing body of literature in the social sciences has investigated the impact of smoking behaviour on the prevalence of obesity with mixed results. Several studies have shown an inverse relationship between smoking and obesity, noting that a decline in smoking can increase body weight and obesity rates [15–18]. However, Fang et al. underlined the absence of any significant impact of smoking on the BMI of obese individuals [18]. The heterogeneity in the effects of smoking on body weight was also highlighted in the recent literature, implying that smoking behaviour may have a different impact across different BMI levels [13, 19].

In addition to the studies showing an inverse association between smoking and body weight, several other studies have generated contradictory findings that indicate a positive relationship between smoking and weight indices [20–22]. Thus, de Oliveira Fonter Gasperin et al. found a positive association between smoking intensity and BMI in current smokers, with heavy smokers being more likely to weigh more than light smokers [23]. Given that both obesity and smoking have severe health effects, potential interactions between them are quite possible. The nature of the relationship between smoking behaviour and body weight still remains unclear, and further evidence should be provided in order to understand the association between them [24].

Recent literature has also attempted to identify whether specific eating habits may lead to a higher risk of obesity. Weight management is mainly determined by energy balance, in which a potential shift to calorie intake that exceeds expenditure may result in higher energy storage and weight gain [25]. A significant number of studies have indicated that an increased intake of energy-dense foods, such as processed meat, fried foods and confections with high sugar content, plays a critical role in the aetiology of overweight and obesity [26–30]. Guallar-Castillón et al. provided a comprehensive explanation of the association between fried foods and obesity and emphasized the relatively low satiety index of fats and the fact that frying usually improves food palatability, which may in turn enhance consumers’ perceived value and satisfaction [31].

Furthermore, processed meat and fried foods are commonly included in convenience meals, such as fast food and ready-to-eat or take-away dishes, which have been extensively suggested as a key obesogenic factor [32–35] due to their high saturated fat and trans fatty acid, processed starch and added sugar content [36, 37]. The perceived convenience of ready-to-eat meals, in combination with a dislike of cooking [38], or even the lack of cooking skills [39], may also contribute to the increase in fast food consumption and its subsequent consequences for diet quality and body weight.

On the other hand, recent evidence has shown that the consumption of whole grain foods improves diet quality [40] and is inversely linked with measures of obesity [41, 42]. In addition, eating patterns that included higher intakes of fruits and vegetables were found to enhance satiety and hinder weight gain [43, 44]. However, as several studies noted, weight management goals can be better achieved when low energy-dense foods (e.g., fruits and vegetables) are included in individuals’ diets as a substitute for energy dense and high-fat foods [29, 45, 46]. Otherwise, simply adding more fruit and vegetable portions without reducing calorie intake from other foods may lead to a higher energy intake and body weight increase [44]. In a recent study, Vernarelli et al. showed that a greater proportion of fruits and vegetables, either as a percentage of total energy or as a percentage of the total weight of food consumed, may decrease the likelihood of being overweight or obese [29].

To curb the obesity epidemic, the identification of the factors that affect body weight can be the cornerstone of interventions to prevent obesity and improve overall health. Under this premise, the present study places a special emphasis on exploring the influence of smoking on body weight. The objectives of this study also include the investigation of the impact of food consumption patterns on BMI and the development of overweightness and obesity, after adjusting for various sociodemographic characteristics.

## Methods

### Sampling procedure

The data employed in this study were drawn from the 2014 German General Social Survey (ALLBUS 2014) on the social monitoring of trends in behaviour, attitudes and social change. German-speaking consumers from both western and eastern Germany who resided in private households and were born before January 1996 were considered as potential survey participants. Participation was voluntary, and individual level data were collected via a formal standardized questionnaire administered in personal interviews and two additional self-completion questionnaires [47]. The data selection procedure was approved by GESIS-Leibniz Institute for the Social Sciences. This study was also given ethical approval by the Ethics Committee of the Democritus University of Thrace.

The sampling procedure was completed in two stages and consisted of probability sampling techniques. In the first stage, a probability proportional to the adult population size was applied to select municipalities in both western and eastern Germany. With respect to the primary sampling points, 111 sample points in 103 western German municipalities and 51 sample points in 45 eastern German municipalities were chosen. In the second stage, individual adult residents were randomly picked up from the municipal registers. A more analytical description of the sampling procedure is provided by Wasmer et al. [48]. Finally, 3471 completed questionnaires were selected [49].

### Measures

Respondents were asked to report their height and current weight. Body weight indices were measured by calculating Body Mass Index (BMI) as body weight in kilograms divided by height square in metres. The World Health Organization (WHO) defines 18.5, 25 and 30 as the BMI cut-offs delineating normal weight, overweight and obesity among adults, respectively [50]. According to the WHO, overweight and obesity are defined as abnormal or excessive fat accumulation that presents health risks [50]. In this study, BMI was assessed through a four-point ordinal scale, taking the value of 1 for underweight respondents and the value of 2 for normal weight. The values of 3 and 4 corresponded to overweight and obese individuals, respectively. Participants were also asked if they smoke. Smoking status was expressed by the smoking participation indicator taking the value of 1 for smokers and 0 for non-smokers. Since there was no further information given on the duration of the smoking habit, all participants who gave a positive response to the introductory question were considered as regular smokers.

Furthermore, a multi-item scale was designed to assess individual’s food consumption patterns. Seven indicators were included in this scale to measure consumption frequencies of specific food groups, such as wholegrain bread (including multigrain bread, wholegrain and multigrain rolls), white-toasted bread (white bread and rolls), fresh fruit, fresh and frozen vegetables, meat and processed meat products (e.g., sausages), deep fried foods (e.g., chips and crisps), and confectionery (sweets, cakes, biscuits, pastries). These indicators were constructed for GESIS-General Social Surveys and mapped basic nutritional categories [49, 51]. All the items were scored on a seven-point frequency scale ranging from several times a day to never (several times a day, every day/almost every day, several times a week, about once a week, two or three times a month, once a month or less often, or never). In the context of the present study, the seven categories of the scale were collapsed into two categories with the frequency of “at least daily” to be the cut-off point indicating frequent consumption of wholegrain and multigrain bread and rolls, white bread and rolls, and fruit and vegetables [52, 53]. In the case of meat products, fried foods, and confectionery, frequent consumption of these food groups was defined as “at least several times a week” [53].

The explanatory variables also pertained to socio-demographic characteristics, specified by dichotomous indicators representing gender, age (18–29 years old, 30–60 years old, or older than 60 years old), marital status (divorced/widowed; married, including registered partnership; or single), educational attainment (up to secondary education; post-secondary education, including the short cycle tertiary level; bachelor level; or postgraduate education, including MSc and PhD) and area of residence (big city, town, or rural area/village).

### Empirical strategy

This study focuses on exploring the impact of cigarette smoking and food consumption patterns on body weight. To assess the influence of smoking on BMI, an important methodological issue that has to be acknowledged is that smoking may be endogenous [18]. If there is endogeneity, smoking estimates may be biased and may not reflect the true effect of smoking status on body weight. Endogeneity can be caused by i) reverse causality (simultaneity bias), ii) omitted variables bias and iii) measurement errors [54]. Given that both obesity and smoking have severe health effects, potential interdependence between them is quite possible. Several studies have indicated statistically significant associations between smoking, body weight and weight management. Smokers may use smoking as a weight control strategy and decide to smoke in order to counteract the effects of overeating, or vice versa [9–11]. In the latter case, smokers may choose a more restricted diet to compensate for the health impacts of smoking. Furthermore, omitted variable bias may stem from unobserved characteristics, such as depression and risk aversion, which may affect smoking and body weight simultaneously [18, 55, 56]. Endogeneity bias may also be attributed to measurement errors, which can be caused by the self-reported measures used in this study to describe individuals’ height, weight and smoking status. To account for potential endogeneity, we adopt the endogenous treatment effects model with an ordered outcome using a latent factor framework [57–59] and estimate the parameters using likelihood simulated techniques [57, 60]. The mathematical expression of our methodology is analytically presented in the Appendix.

Under this approach, the outcome of undertaking the treatment (i.e., smoking participation) is explained by an ordered discrete outcome described by body weight status. The outcome equation, as expressed by BMI classification, includes a vector of explanatory variables depicting respondents’ sociodemographic characteristics, such as gender, age, marital status, the population size of the area of residence, eating habit indicators, and the treatment variable (smoker, non-smoker).

In addition to the explanatory variables included in the outcome equation, the treatment equation (smoking participation) also encompasses exogenous variables to acknowledge potential endogeneity. In particular, education is supposed to be directly associated with smoking behaviour but seems to have no direct effect on body weight [61]. Furthermore, we instrument smoking participation with smoking prevalence at individuals’ state of origin [62]. Community-level smoking prevalence has been widely used as an instrumental variable because it can have a key role in the decision to smoke by influencing attitudes about smoking, but it is unlikely to have a direct effect on BMI [18].

All analyses are performed separately for males and females in order to investigate potential gender differences in the impact of the main variables of interest on body weight. For the sake of comparison, the univariate ordered probit model is also employed with no assumption for endogeneity issues [63]. Furthermore, in order to provide a deeper insight into the influence of smoking on body weight, the marginal treatment effects (MTE) are estimated [57, 58]. The MTE are expressed as the average effect of smoking on BMI for individuals who are on the margin of indifference between having decided to smoke or not.

## Results

Table 1 provides detailed information about the sample characteristics. For the ALLBUS sample, 44% of the participants were considered to be of normal weight, 36% were overweight and 18% conformed to the clinical definition of obesity, whereas the rest of the sample was classified as underweight. The majority of participants were non-smokers, as 71.2% reported zero cigarette consumption.

**Table 1:** Characteristics of the study participants

Variables	Total (*N* = 3471)	Males (*N* = 1762)	Females (*N* = 1709	Chi-square	*p*-value
Age				3.157	0.206
18–29 years old	583 (16.8%)	308 (17.5%)	275 (16.1%)		
30–60 years old	1899 (54.8%)	938 (53.3%)	961 (56.3%)		
Older than 60 years	986 (28.4%)	514 (29.2%)	472 (27.6%)		
Marital status				**77.825**	**0.000**
Married	1998 (57.6%)	1039 (59.0%)	959 (56.2%)		
Divorced/widowed	496 (14.3%)	165 (9.4%)	331 (19.4%)		
Single	973 (28.1%)	556 (31.6%)	417 (24.4%)		
Area of residence				2.660	0.264
Big city	1104 (31.8%)	564 (32.0%)	540 (31.6%)		
Town	1006 (29.0%)	490 (27.8%)	516 (30.2%)		
Village/Rural area	1360 (39.2)	708(40.2%)	652 (38.2%)		
Educational attainment				**12.879**	**0.002**
Up to secondary education	1993 (57.5%)	973 (55.3%)	1020 (59.8%)		
Post-secondary education	767 (22.1%)	386 (21.9%)	381 (22.3%)		
Postgraduate studies	706 (20.4%)	400 (22.7%)	306 (17.9%)		
Frequent consumption of:					
Whole grain bread	2096 (60.4%)	982 (55.8%)	1114 (65.2%)	**31.961**	**0.000**
White-toasted bread	983 (28.3%)	581 (33.0%)	402 (23.5%)	**38.180**	**0.000**
Fruits	2305 (66.4%)	995 (56.5%)	1310 (76.7%)	**157.917**	**0.000**
Fresh-frozen vegetables	1534 (44.2%)	621 (35.3%)	913 (53.4%)	**115.954**	**0.000**
Meat products	2882 (83.1%)	1590 (90.3%)	1292 (75.6%)	**132.982**	**0.000**
Deep fried foods	274 (7.9%)	188 (10.7%)	86 (5.0%)	**37.982**	**0.008**
Confectionery	1957 (56.4%)	949 (53.9%)	1008 (59.0%)	**9.145**	**0.002**
BMI				**94.948**	**0.000**
Underweight	67 (2.0%)	14 (0.8%)	53 (3.2%)		
Normal weight	1503 (44.0%)	663 (37.8%)	840 (50.5%)		
Overweight	1230 (36.0%)	735 (41.9%)	495 (29.7%)		
Obese	619 (18.0%)	342 (19.5%)	277 (16.6%)		
Smoking behaviour					
Smoker (Smoking participation)	998 (28.8%)	586 (33.3%)	412 (24.1%)	**35.717**	**0.000**
				**t-test**	**p-value**
Smoking prevalence^a^	25.365 (5.236)	30.005 (2.492)	20.581 (2.046)	**62.193**	**0.000**

^a^Mean, standard deviation in parentheses

(Statistically significant variables are highlighted in bold)

Non-parametric tests (chi-square) showed a statistically significant association of gender with BMI, smoking and food consumption patterns. Furthermore, more women than men reported frequent consumption of whole grain bread, fruits, and fresh and frozen vegetables. On the other hand, the percentage of male respondents that declared frequent consumption of less healthy food items, such as white bread, meat products and deep-fried foods, was higher than that of females.

For comparison, we provide the estimates of both the endogenous treatment effects model with an ordered outcome (as described above) and the univariate ordered probit model. In the latter approach, we assumed the absence of endogeneity between smoking and BMI. Table 2 shows the univariate ordered probit model estimations, in which the smoking indicator was found to be statistically insignificant for both male and female participants*,* suggesting that smoking behaviour has no considerable influence on body weight.

**Table 2:** BMI estimates of the univariate ordered probit model with no assumption of endogeneity between smoking and body weight

Variables	BMI		
	Full sample	Males	Females
Smoker (smoking participation)	− 0.058	− 0.089	− 0.022
	(0.045)	(0.060)	(0.068)
Sociodemographic characteristics^a^			
Gender (women)	−0.282***	–	–
	**(0.041)**		
30–60 years old	0.441***	0.446***	0.437***
	**(0.064)**	**(0.089)**	**(0.092)**
Over 60 years old	0.608***	0.508***	0.696***
	**(0.075)**	**(0.105)**	**(0.107)**
Married	0.167***	0.213***	0.141*
	**(0.054)**	**(0.074)**	**(0.080)**
Divorced/widowed	0.213***	0.157	0.209**
	**(0.072)**	(0.110)	**(0.098)**
Big city	−0.154***	−0.128*	−0.175**
	**(0.049)**	**(0.069)**	**(0.071)**
Village/Rural area	0.060	0.047	0.091
	(0.047)	(0.066)	(0.067)
Frequent consumption of:			
Whole grain bread	−0.029	−0.088	0.041
	(0.040)	(0.056)	(0.060)
White/toasted bread	−0.053	− 0.134**	0.044
	(0.043)	**(0.058)**	(0.066)
Fruits	−0.023	−0.044	0.017
	(0.045)	(0.060)	(0.071)
Vegetables	−0.023	0.031	−0.063
	(0.042)	(0.060)	(0.059)
Meat products	0.299***	0.364***	0.254***
	**(0.054)**	**(0.093)**	**(0.066)**
Deep fried foods	−0.016	−0.121	0.160
	(0.074)	(0.091)	(0.132)
Confectionery	−0.159***	−0.191***	− 0.133**
	**(0.039)**	**(0.055)**	**(0.056)**
μ_1_	−1.742	−1.984	−1.287
μ_2_	0.359	0.280	0.768
μ_3_	1.428	1.483	1.690
Log-Likelihood	− 3644.031	− 1835.938	− 1780.192

Standard errors are given in parentheses

^a^Age: 18–29 years old (reference category), Marital status: single (reference category)

Area of residence: town (reference category)

*p < 0.1, **p < 0.05, ***p < 0.01 (Statistically significant variables are highlighted in bold)

The likelihood ratio test performed to assess the independence between the smoking equation and BMI equation in the endogenous treatment effects model for the male subsample rejected the null hypothesis that there is no correlation between the treatment errors and the outcome errors (chi-square = 9.030, *p <* 0.01). Furthermore, the likelihood ratio test applied for the subsample of female participants revealed that there is a significant correlation between the treatment errors and the outcome errors (chi-square = 4.090, *p <* 0.05). Thus, taking potential endogeneity issues into consideration is a crucial aspect of ensuring that the analysis procedure culminates in unbiased estimates of smoking’s effects on individuals’ body weight.

Tables 3 and 4 provide the estimations of the treatment and outcome equations of the endogenous treatment effects model approach, respectively. First, smoking participation determinants are presented in Table 3. The instrumental variables adopted for the analysis were statistically significant with a strong predictive power. In particular, smoking prevalence had an important impact on the decision to smoke in both genders (full sample: γ = 0.061, *p* < 0.01, males: γ = 0.064, p < 0.01, females: γ = 0.056, *p* < 0.05). In the same manner, educational attainment was also significantly associated with the decision to smoke, with respondents who reported receiving post-secondary education (full sample: γ = − 0.497, p < 0.01) or undertaking postgraduate studies (full sample: γ = − 0.782, *p* < 0.01) being less likely to smoke than participants with a lower educational level. Similar results were obtained for both males and females.

**Table 3:** Smoking status estimates (treatment equation) of the endogenous treatment effects model with an ordered outcome considering potential endogeneity between smoking and body weight

Variables	Smoking participation		
	Full sample	Males	Females
Sociodemographic characteristics^a^			
Gender (women)	0.288*	–	–
	**(0.152)**		
30–60 years old	0.283***	0.346**	0.195
	**(0.111)**	**(0.148)**	(0.164)
Over 60 years old	− 0.849***	−0.748***	− 0.999***
	**(0.137)**	**(0.185)**	**(0.206)**
Married	−0.283***	−0.186	− 0.384***
	**(0.095)**	(0.127)	**(0.143)**
Divorced/widowed	0.137	0.302	0.043
	(0.128)	(0.187)	(0.179)
Big city	0.026	0.031	−0.007
	(0.088)	(0.121)	(0.130)
Village/Rural area	−0.182**	−0.218*	− 0.153
	**(0.082)**	**(0.113)**	(0.123)
Post-secondary education	−0.497***	− 0.522***	− 0.486***
	**(0.082)**	**(0.111)**	**(0.126)**
Post-graduate studies	−0.782***	− 0.855***	− 0.732***
	**(0.092)**	**(0.117)**	**(0.155)**
Frequent consumption of:			
Whole grain bread	−0.170**	− 0.124	− 0.229**
	**(0.071)**	(0.095)	**(0.108)**
White-toasted bread	0.189**	0.353***	−0.057
	**(0.078)**	**(0.101)**	(0.124)
Fruits	−0.468***	−0.557***	− 0.383***
	**(0.079)**	**(0.103)**	**(0.124)**
Vegetables	−0.124*	0.137	−0.422***
	**(0.075)**	(0.104)	**(0.108)**
Meat products	0.299***	0.349**	0.309**
	**(0.097)**	**(0.160)**	**(0.124)**
Deep fried foods	0.119	0.157	0.102
	(0.126)	(0.151)	(0.228)
Confectionery	−0.180***	−0.182*	−0.216**
	**(0.069)**	**(0.094)**	**(0.103)**
Smoking prevalence	0.061***	0.064***	0.056**
	**(0.014)**	**(0.018)**	**(0.025)**
Log-Likelihood function	− 1797.997	− 975.637	− 808.419

Standard errors are given in parentheses

^a^Age: 18–29 years old (reference category), Marital status: single (reference category)

Area of residence: town (reference category), Educational attainment: up to secondary education (reference category)

*p < 0.1, **p < 0.05, ***p < 0.01 (Statistically significant variables are highlighted in bold)

**Table 4:** BMI estimates (outcome equation) of the endogenous treatment effects model with an ordered outcome considering potential endogeneity between smoking and body weight

Variables	BMI		
	Full sample	Males	Females
Smoker (smoking participation)	1.155***	1.236***	0.634*
	**(0.335)**	**(0.439)**	**(0.332)**
Sociodemographic characteristics^a^			
Gender (women)	0.329***	–	–
	**(0.070)**		
30–60 years old	0.536***	0.554***	0.463***
	**(0.110)**	**(0.166)**	**(0.112)**
Over 60 years old	1.022***	0.948***	0.879***
	**(0.191)**	**(0.258)**	**(0.170)**
Married	0.332***	0.418***	0.206**
	**(0.101)**	**(0.153)**	**(0.102)**
Divorced/widowed	0.261**	0.158	0.222*
	**(0.114)**	(0.180)	**(0.118)**
Big city	−0.198***	−0.169	−0.188**
	**(0.075)**	(0.110)	**(0.081)**
Village/Rural area	0.124*	0.130	0.116
	**(0.071)**	(0.107)	(0.079)
Frequent consumption of:			
Whole grain bread	0.014	−0.075	0.080
	(0.059)	(0.087)	(0.070)
White-toasted bread	−0.158**	−0.337***	0.040
	**(0.073)**	**(0.122)**	(0.078)
Fruits	0.107	0.115	0.077
	(0.075)	(0.108)	(0.093)
Vegetables	0.014	0.008	−0.005
	(0.061)	(0.091)	(0.070)
Meat products	0.318***	0.408***	0.234***
	**(0.083)**	**(0.153)**	**(0.076)**
Deep fried foods	−0.067	−0.216	0.140
	(0.107)	(0.147)	(0.156)
Confectionery	−0.160***	−0.217**	− 0.113**
	**(0.058)**	**(0.089)**	**(0.065)**
μ_1_	−1.805	−2.273	−1.135
μ_2_	1.033	0.946	1.142
μ_3_	2.495	2.689	2.171
Log-Likelihood (both stages)	− 5423.587	− 2800.598	− 2584.326

Standard errors are given in parentheses

^a^Age: 18–29 years old (reference category). Marital status: single (reference category)

Area of residence: town (reference category)

*p < 0.1. **p < 0.05. ***p < 0.01 (Statistically significant variables are highlighted in bold)

Table 3 also provides insights into the relationships between eating patterns and smoking behaviour. More specifically, female smokers were less likely to consume whole grain and multigrain bread (γ = − 0.229, *p* < 0.05), fruits (γ = − 0.383, *p* < 0.01), confectionery (γ = − 0.216, *p* < 0.05), and fresh/frozen vegetables (γ = − 0.422, *p* < 0.01) on a frequent basis. In male smokers, a frequent consumption of fruits (γ = − 0.557, p < 0.01) and confectionery (γ = − 0.182, *p* < 0.10) was inversely linked with smoking. For both genders, the regular consumption of meat products showed an upward trend in smokers, whereas male smokers were also more likely to be frequent consumers of white-toasted bread (Table 3).

Table 4 displays the estimations derived from the outcome regression of the endogenous treatment effects model. The estimated parameters of smoking were statistically significant and had a positive sign, implying that both male and female smokers have a greater likelihood of increased BMI (males: *β* = 1.236, *p* < 0.01, females: *β* = 0.634, *p* < 0.10). In comparison with the smoking indicator estimates from the univariate ordered probit model in Table 2, the endogenous treatment effects model indicates that smokers are more likely to have a higher body weight than non-smokers (Table 4). Thus, if potential endogeneity is ignored, the coefficient of smoking will not reflect the true effect of smoking on body weight.

With regard to eating habits, several regressors were found to have statistical significance in the analysis. Frequent consumption of white-toasted bread was inversely linked with the BMI of male participants (*β* = − 0.337, *p* < 0.01). Contrary to our anticipation, BMI was also negatively correlated with frequent confection consumption in both genders (males: *β* = − 0.217, *p* < 0.05, females: *β* = − 0.113, *p* < 0.10). On the other hand, individuals who reported consuming meat at least several times a week were more likely to have a higher BMI (males: *β* = 0.408, *p* < 0.01, females: *β* = 0.234, *p* < 0.01).

The estimated MTEs presented in Table 5 for the male subsample show that smoking decreases the probability of being underweight by 2.2% and having a normal weight by 24.5%, while it increases the probability of being obese by 23.6%. Furthermore, female smokers are 17.2% less likely to have a healthy weight, and in comparison with men, they are less likely to be obese (15.1% vs 23.6%).

**Table 5:** Marginal treatment effects of smoking on BMI (average effect of smoking participation on BMI category)

BMI categories	Full sample	Males	Females
Underweight	−**0.037**	**− 0.022**	**− 0.034**
	[− 0.173, 0.000]	[−0.118, 0.000]	[−0.134, 0.000]
Normal weight	−**0.243**	**−0.245**	**− 0.172**
	[−0.273, 0.000]	[−0.278, 0.000]	[−0.196, 0.000]
Overweight	0.049	0.032	0.054
	[−0.121, 0.172]	[− 0.133, 0.185]	(−0.050, 0.112]
Obese	**0.231**	**0.236**	**0.151**
	[0.000, 0.310]	[0.000, 0.308]	[0.000, 0.217]

^a^Confidence intervals are reported in brackets

(Statistically significant estimates of smoking are highlighted in bold)

## Discussion

This study showed that addressing endogeneity issues is of critical importance for obtaining unbiased estimates of the impact of smoking on body weight. Ignoring potential endogeneity may affect both the statistical significance of the smoking estimates and the direction of influence of smoking on BMI. Our findings showed that smoking is positively associated with BMI, with smokers presenting a much higher likelihood of obesity and a lower likelihood of being within a healthy weight range than non-smokers. The influence of smoking on body weight also seems to be stronger in male participants, since male smokers have a higher probability of being obese compared to female smokers.

Our findings raised questions about the common belief pertaining to the efficacy of smoking as a weight control strategy [11]. A recent study also noted that smokers gained more weight than non-smokers over a 2-year period, reaching the conclusion that even light smoking may be correlated with subsequent weight gain [64]. However, the results of previous studies on the association between smoking and body weight remain inconclusive. A significant body of literature has indicated that smoking and BMI are inversely related [13, 18, 19, 22], whereas other studies ended up finding a negligible association between smoking status and obesity [23, 61].

One plausible explanation for this increasing trend in obesity rates in smokers may be the fact that smoking can lead to lower physical activity levels by restricting respiratory function [13, 14]. On a very consistent basis, body weight increase seems to be an aftereffect of physical inactivity, indicating that public health efforts to prevent obesity should pay attention to the enhancement of physical exercise and a more active lifestyle among adults [65].

In addition, the positive influence of cigarette smoking on BMI may be attributed to the dietary patterns of smokers. In line with recent research indicating less healthy eating habits for smokers [66, 67], our study showed that smokers’ eating habits are characterized by frequent intake of more energy-dense foods (e.g., meat products and white-toasted bread) and less frequent consumption of healthy food items, such as whole-grain bread, vegetables and fruits. These food choices may result in a poor diet and subsequent weight gain. Furthermore, it should be noted that additional factors may influence both smoking status and BMI but were not included in the analyses due to the lack of data. For instance, stress and loss of employment constitute factors that are positively correlated with both smoking and body weight [68].

With respect to eating patterns, several indicators describing frequent consumption of various food groups were found to have a significant relationship with body weight status. In agreement with recent studies, frequent consumption of meat products was positively related with individuals’ BMI in both genders [26, 27]. Meat, and especially processed meat, is commonly included in convenience foods, which, due to their high calorie content, may contribute to body weight increase [32, 33]. Given that meat products are common in Western dietary patterns, a decrease in their intake may prompt a subsequent decrease in the prevalence of overweightness and obesity. On the other hand, a daily consumption of white-toasted bread was less likely to lead to a BMI increase in men. Although it is commonly believed that bread consumption is positively related with the risk of obesity, this topic still remains controversial [69]. According to Loria-Kohen et al., the consumption of common foods, such as bread, may offer a greater ease of following a well-balanced diet and avoiding high-fat foods. Bread consumption can also increase the sensation of satiety and result in a better compliance with diet and even fewer dropouts in weight-loss diets [70].

Contrary to our anticipation, frequent confectionery consumption was found to be inversely associated with BMI status. A possible explanation for this relationship may be the potential underreporting of actual intake. The extent of underreporting has been shown to be positively related to one’s BMI, indicating that overweight and obese individuals are more likely to underreport food consumption [71, 72]. The consumption of high-fat and high-sugar foods has also been linked with underreporting [72]. Therefore, it seems that overweight individuals are more likely to underreport their actual confectionery consumption. Furthermore, individuals who reported frequent confectionery consumption may prefer small portions in order to avoid weight gain. Otherwise, a rare consumption could increase the desire for sweets, resulting in overconsumption or larger portion sizes. Frequent consumption of fruit and vegetables was found to have a negligible impact on BMI. Although several studies have shown that the consumption of fruits and vegetables is inversely correlated with measures of obesity [73], a significant amount of research provides inconsistent results [44, 74]. The latter studies underline the need to counteract the effect of a higher caloric intake, which may result after an increase in fruit and vegetable consumption, by restricting the intake of more energy-dense foods in order to avoid a positive energy balance and subsequent weight gain.

## Limitations

Some limitations of this study have to be acknowledged. First, the cross-sectional design cannot infer causality. Future research employing longitudinal designs would help to explore potential causal associations. Second, this study employs self-reported data on individuals’ weight and height, smoking status and food consumption frequencies. The use of self-reporting may result in over- or under- reporting due to limited recall, social desirability or other biases [75]. Although BMI and self-reported smoking are considered to be good proxies for body weight and smoking, respectively [11], future studies should consider the inclusion of additional body weight and smoking status measures. Furthermore, the food consumption measures may not represent usual food consumption patterns, which shape individuals’ weight status. For instance, an overweight individual might have been following a restricted diet programme at the time of the survey.

## Conclusions

By addressing the endogenous selection of smoking, estimations revealed that smoking is positively associated with BMI, with smokers being at an increased risk of obesity and presenting a lower likelihood to be within a healthy weight range. Furthermore, the impact of smoking on the risk of obesity is stronger in male participants compared to female smokers. Our findings challenge the general belief about the role of smoking as an effective weight control strategy. Therefore, health instructors may proceed with anti-smoking policies without suspicions of potential weight gain in overweight and obese smokers. Food consumption frequency is also found to influence body weight, although potential underreporting, especially among overweight individuals, should be considered in future research. Smoking behaviour is linked with less healthy food choices and poor diet quality, which may lead to weight gain and a subsequent increase in overweightness and obesity rates in smokers. Our results highlight the necessity of properly designed and implemented health strategies to decrease the prevalence of both smoking and obesity since both outcomes seem to be interdependent. Health interventions and nutrition programmes should also be tailored according to the specific characteristics of different consumer groups in order to promote a healthy lifestyle and introduce successful weight loss tactics.

## Appendix

### Econometric framework

To address potential endogeneity issues, we employed the endogenous treatment effects model with an ordered outcome, as introduced by Gregory [36]. The outcome of undertaking the treatment (i.e., smoking participation) is explained by an ordered discrete variable expressed through BMI, classified in ascending order from underweight to obese. Therefore, the outcome equation can be specified as [36, 37]:

1 $$ {Y}_i=\left\{\begin{array}{c}\ 1\kern1em if\kern0.75em -\infty <{Y}_i^{\ast }<{\mu}_1\\ {}2\kern0.75em if\kern1.25em {\mu}_1\le {Y}_i^{\ast }<{\mu}_2\\ {}.\\ {}.\\ {}.\\ {}J\kern1em if\kern1em {\mu}_J\le {Y}_i^{\ast }<\infty \end{array}\right\}, $$

where μ_1_, μ_2_,…, μ_J_ represent threshold parameters to be estimated, j = 1,2,…J delineate possible BMI categories J and ${Y}_i^{\ast }$ is the latent outcome variable for the i^th^ participant expressed as:

2 $$ {Y}_i^{\ast }={\boldsymbol{Q}}_{\boldsymbol{i}}{\beta}_1+{T}_i{\beta}_2+{\varepsilon}_{i.\kern0.5em } $$

In our model specification, *ε*_*i*_ is the error term, ***Q***_***i***_is a vector of explanatory variables, and *Τ*_*i*_ represents the treatment variable corresponding to respondents’ smoking behaviour as follows:

3 $$ {T}_i=\left\{\begin{array}{c}1\kern1em if\kern1em {\boldsymbol{Q}}_{\boldsymbol{i}}{\gamma}_1+{\boldsymbol{Z}}_{\mathbf{1}\boldsymbol{i}}{\gamma}_2+{Z}_{2i}{\gamma}_3+{u}_i>0\\ {}\ 0\kern1em if\kern1em {\boldsymbol{Q}}_{\boldsymbol{i}}{\gamma}_1+{\boldsymbol{Z}}_{\mathbf{1}\boldsymbol{i}}{\gamma}_2+{Z}_{2i}{\gamma}_3+{u}_i\le 0\kern0.75em \end{array}\right\}, $$

where *T*_*i*_ indicates the decision to smoke taking the value of 1 for smokers and 0 for non-smokers, and *u*_*i*_ is the error term. To address concerns about omitted variable bias and reverse causality, exogenous variables, ***Z***_**1*****i***_ and *Ζ*_2*i*_, are included in the treatment equation. In particular, ***Z***_**1*****i***_ is a vector of three indicators used in the analysis to describe individual’s educational attainment. We also instrument smoking participation with smoking prevalence at individuals’ state of origin (*Ζ*_2*i*_).

To address potential violations of joint normality in the error terms, *u*_*i*_ and *ε*_*i*_, a latent factor framework is adopted where unobservables in the treatment and outcome equations are generated by a factor structure [36, 38]. Thus, the error terms, *u*_*i*_ and *ε*_*i*_, in eqs. (2) and (3) are supposed to have the following factor structure:

4 $$ {u}_{i=}{\lambda}_T{\eta}_i+{\zeta}_i $$

5 $$ {\varepsilon}_i={\lambda}_Y{\eta}_i+{\iota}_{i.} $$

where the marginal distributions of *ζ* and *ι* are assumed to be normal and *λ*_τ,_ *λ*_Υ_ are loading factors that indicate the dependence between the unobservables in the treatment and outcome equations.

Finally, we estimate the parameters in eqs. (1)–(3) using likelihood simulated techniques [36, 39]. Therefore, the distribution of the latent factor, *η*, is approximated by taking quasi-random draws based on Halton sequences from its chosen distribution, and then entered into the model such as observed covariates. For reasons of simplicity in presentation, we set ***Q***_***i***_*γ*_1_ + ***Z***_**1*****i***_*γ*_2_ + *Ζ*_2*i*_*γ*_3_=***L***_***i***_*γ* and ***Q***_***i***_*β*_1_ + *Τ*_*i*_*β*_2_=***X***_***i***_*β*. Thus, the likelihood function can be assessed as follows:

$$ {L}_i=\frac{1}{S}\prod \limits_{i=1}^N\sum \limits_{s=1}^S\varPhi \left\{\tau \times \left({\boldsymbol{L}}_{\boldsymbol{i}}\gamma +{\lambda}_T{\eta}_i\right)\right\}\times \sum \limits_{k=1}^K\left\{I\times \left({Y}_i=k\right)\right\}\left\{\varPhi \left({\mu}_k-{\boldsymbol{X}}_{\boldsymbol{i}}\beta +{\lambda}_{\gamma }{\eta}_i\right)-\varPhi \left({\mu}_{k-1}-{\boldsymbol{X}}_{\boldsymbol{i}}\beta +{\lambda}_{\gamma }{\eta}_i\right)\right\},(6) $$

where S is the number of simulation draws, *Φ* is the standard normal distribution, *I* stands for an indicator function, τ = 2 × *T*_*i*_˗1 and k = J + 1.

## Abbreviations

BMI: Body Mass Index
WHO: World Health Organization
